# Serum apolipoprotein A-I is a novel prognostic indicator for non-metastatic nasopharyngeal carcinoma

**DOI:** 10.18632/oncotarget.5823

**Published:** 2015-10-19

**Authors:** Xiao-Lin Luo, Guang-Zheng Zhong, Li-Yang Hu, Jie Chen, Ying Liang, Qiu-Yan Chen, Qing Liu, Hui-Lan Rao, Kai-Lin Chen, Qing-Qing Cai

**Affiliations:** ^1^ Department of Gynecologic Oncology, Sun Yat-sen University Cancer Center, State Key Laboratory of Oncology in South China, Collaborative Innovation Center for Cancer Medicine, Guangzhou, China; ^2^ Department of Urology, Sun Yat-sen Memorial Hospital, Sun Yat-sen University, Guangzhou, China; ^3^ Department of Medical Oncology, Sun Yat-sen University Cancer Center, State Key Laboratory of Oncology in South China, Collaborative Innovation Center for Cancer Medicine, Guangzhou, China; ^4^ Guangdong Province Key Laboratory of Arrhythmia and Electrophysiology, Radiotherapy Department, Sun Yat-sen Memorial Hospital, Sun Yat-sen University, Guangzhou, China; ^5^ Department of Nasopharyngeal Carcinoma, Sun Yat-sen University Cancer Center, State Key Laboratory of Oncology in South China, Collaborative Innovation Center for Cancer Medicine, Guangzhou, China; ^6^ Department of Cancer Prevention Research, Sun Yat-sen University Cancer Center, State Key Laboratory of Oncology in South China, Collaborative Innovation Center for Cancer Medicine, Guangzhou, China; ^7^ Department of Pathology, Sun Yat-sen University Cancer Center, State Key Laboratory of Oncology in South China, Collaborative Innovation Center for Cancer Medicine, Guangzhou, China

**Keywords:** non-metastatic nasopharyngeal carcinoma (NPC), apolipoprotein A-I (ApoA-I), prognosis

## Abstract

**Background:**

We investigated the value of pretreatment serum apolipoprotein A-I (ApoA-I) in complementing TNM staging in the prognosis of non-metastatic nasopharyngeal carcinoma (NPC).

**Patients and methods:**

We retrospectively reviewed 1196 newly diagnosed patients with non-metastatic NPC. Disease-specific survival (DSS), distant metastasis-free survival (DMFS), and locoregional recurrence-free survival (LRFS) rates were compared according to serum ApoA-I level. Multivariate analysis was performed to assess the prognostic value of serum ApoA-I.

**Results:**

The 5-year DSS, DMFS, and LRFS rates for patients with elevated or decreased serum ApoA-I were 81.3% versus 69.3% (*P* < 0.001), 83.4% versus 67.4% (*P* < 0.001), and 80.9% versus 67.3% (*P* < 0.001), respectively. ApoA-I ≥ 1.025 g/L was an independent prognostic factor for superior DSS, DMFS, and LRFS in multivariate analysis. After stratification by clinical stage, serum ApoA-I remained a clinically and statistically significant predictor of prognosis.

**Conclusion:**

Our data suggest that the level of ApoA-I at diagnosis is a novel independent prognostic marker that could complement clinical staging for risk definition in non-metastatic NPC.

## INTRODUCTION

Nasopharyngeal carcinoma (NPC) is a unique malignancy with a remarkable geographic distribution, which occurs with much greater frequency in Southern China [1, 2]. The recent reported incidence of NPC in Southern China is 30–80 per 100,000 people per year [3]. The current standard regimen is radiotherapy (RT) alone for early-stage disease and concurrent chemoradiotherapy (CRT) for locally advanced disease [1]. Although NPC is mostly radiosensitive, 20–30% of patients ultimately develop distant metastasis and/or recurrence despite of treatment [4]. Currently, the prognosis for NPC patients is mainly based on TNM staging system. However, patients with the same TNM stage and similar treatment regimens often present with variable clinical outcomes, suggesting that the present staging system is not adequate for prognosis. Therefore, it is critical to define reliable prognostic factors to complement the TNM staging system to identify high-risk patients for combined and aggressive treatment.

It is reported that lipid metabolism plays an important role in the pathogenesis and progression of cancer [5–8]. Increased serum cholesterol level is associated with an increased risk for certain cancers, such as breast cancer, prostate cancer, and colon cancer [9–12]. There is a report that serum levels of triglyceridesare correlated with cancer morbidity [13]. Serum high-density lipoprotein cholesterol (HDL-C) level may be a clinical prognostic factor in gastric and lung cancers [8, 14]. Recent studies have suggested that ApoA-I, the major protein constituent of HDL-C, is a prognostic biomarker for ovarian, breast and pancreatic cancers [15–18]. Recent retrospective studies have revealed that elevated ApoA-I level is significantly associated with favorable prognosis in patients with metastatic NPC and non-small-cell lung cancer [19, 20]. However, little is known about the prognostic significance of ApoA-I in patients with non-metastatic NPC.

The aim of this study is to determine the prognostic value of serum ApoA-I in newly diagnosed non-metastatic NPC patients.

## RESULTS

### Patient characteristics

The clinical characteristics of the 1196 patients are shown in Table 1. These patients are men dominated. The median age was 46 years (range 11–80 years). The distribution of disease stage was: stage I, 3.4% (41 patients); stage II, 16.1% (193 patients); stage III, 50.8% (608 patients) and stage IV, 29.6% (354) patients. The mean ApoA-I level was 1.286 g/L (range 0.29–3.1 g/L). The cut-off points of baseline serum lipid and lipoprotein levels were determined by ROC curve analyses (Supplementary Figure S1). An ApoA-I value of 1.025 g/L resulted in the most appropriate sensitivity and specificity for DSS. Using 1.025 g/L as the cutoff point, we identified 183 patients (15.3%) as having decreased ApoA-I. Similarly, a triglyceride value of 1.865 mmol/L, cholesterol value of 5.045 mmol/L, HDL-C value of 1.065 mmol/L, LDL-C value of 5.715 mmol/L, and Apo-B value of 1.880 g/L were selected as the optimal cut-off points for survival analysis. Baseline clinical features of patients with elevated ApoA-I at diagnosis were compared with those of patients with decreased ApoA-I at diagnosis. No significant between-group difference was observed for age, N stage, EA/IgA, and different levels of triglyceride, LDL-C, and Apo-B. Patients with ApoA-I < 1.025 g/L at diagnosis tended to be men, presentingat later clinical stages and T stages, and receiving CRT. Patients with lower ApoA-I at diagnosis usually presented with a higher titer of VCA/IgA, and lower levels of total cholesterol and HDL-C.

**Table 1:** Clinical characteristics of patients according to ApoA-I levels at diagnosis

Characteristics	ApoA-I ≥ 1.025 g/L	ApoA-I < 1.025 g/L	*P* value
	*n* (%)	*n* (%)	
Age, years			0.309
≤45	490 (48.4)	96 (52.5)	
>45	523 (51.6)	87 (47.5)	
Sex			**0.007**
Female	260 (25.7)	30 (16.4)	
Male	753 (74.3)	153 (83.6)	
Clinical stage			**0.029**
I/II	209 (20.6)	25 (13.7)	
III/IV	804 (79.4)	158 (86.3)	
T stage			**0.009**
T1/T2	343 (33.9)	44 (24.0)	
T3/T4	670 (66.1)	139 (76.0)	
N stage			0.888
N0/N1	609 (60.1)	109 (59.6)	
N2/N3	404 (39.9)	74 (40.4)	
VCA-IgA^a^			**0.006**
<1:80	109 (11.2)	8 (4.5)	
≥1:80	864 (88.8)	170 (95.5)	
EA-IgA^b^			0.498
<1:10	134 (14.8)	22 (12.8)	
≥1:10	773 (85.2)	150 (87.2)	
Triglyceride (mmol/L)			0.228
<1.865	840 (82.9)	145 (79.2)	
≥1.865	173 (17.1)	38 (20.8)	
Cholesterol (mmol/L)			**< 0.001**
<5.045	512 (50.5)	122 (66.7)	
≥5.045	501 (49.5)	61 (33.3)	
HDL-C (mmol/L)			**< 0.001**
<1.065	202 (19.9)	138 (75.4)	
≥1.065	811 (80.1)	45 (24.6)	
LDL-C (mmol/L)			0.394
<5.715	1000 (98.7)	182 (99.5)	
≥5.715	13 (1.3)	1 (0.5)	
Apo-B (g/L)			0.415
<1.880	1004 (99.1)	183 (100)	
≥ 1.880	9 (0.9)	0 (0)	
Treatment			**0.030**
CRT	706 (69.7)	142 (77.6)	
RT	307 (30.3)	41 (22.4)	
Diagnosis of diabetes			0.344
Yes	75	10	
No	936	173	
Diagnosis of hypertension			0.781
Yes	219	38	
No	791	345	

Abbreviations: ApoA-I, apolipoprotein A-I; VCA-IgA, immunoglobulin A/virus capsid antigen; EA-IgA, immunoglobulin A/early antigen; HDL-C, high-density lipoprotein cholesterol; LDL-C, low-density lipoprotein cholesterol; Apo-B, apolipoprotein B; CRT, chemoradiotherapy; RT, radiotherapy.

a Complete information on VCA-IgA was available in 1151 cases;

b Complete information on EA-IgA was available in 1079 cases.

### Univariate analysis of ApoA-I as a prognostic factor for DSS, DMFS, and LRFS

**Figure 1:**
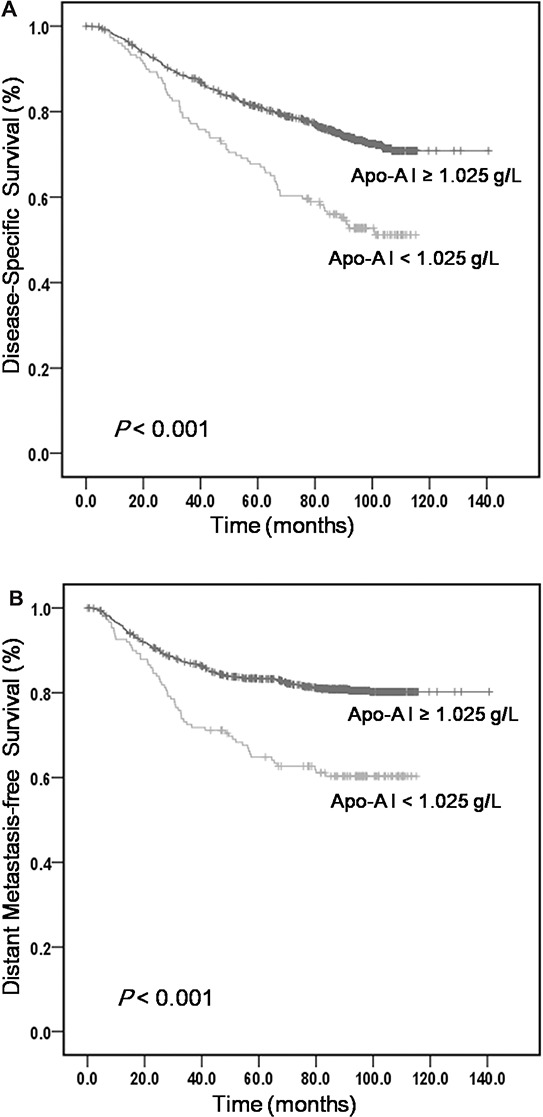

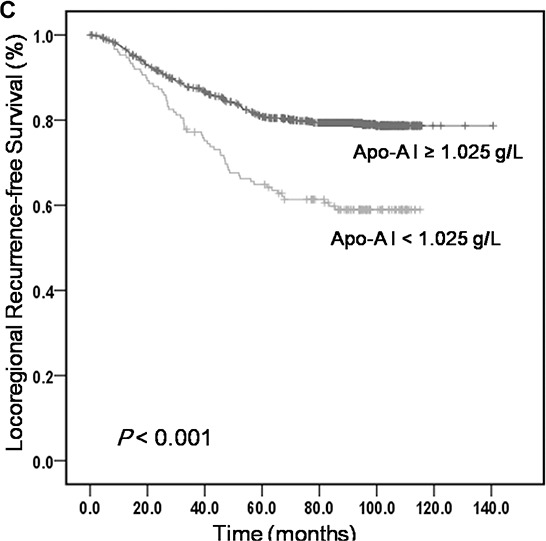
Kaplan-Meier curves obtained from univariate analyses (log-rank) of 1196 patients with NPC based on serum ApoA-I levels **A.** DSS curves based on ApoA-I levels. **B.** DMFS curves based on ApoA-I levels. **C.** LRFS curves based on ApoA-I levels.

The results of univariate analysis are presented in Table 2. Using 1.025 g/L as a cut-off value, serum ApoA-I level was strongly associated with DSS, DMFS, and LRFS. The 5-year estimate for DSS was 81.3% for patients with ApoA-I ≥ 1.025 g/L and 69.3% for those with ApoA-I < 1.025 g/L (*P* < 0.001; Figure 1A). Patients with ApoA-I < 1.025 g/L at diagnosis also had worse 5-year DMFS (83.4% vs. 67.4%, *P* < 0.001; Figure 1B) and worse 5-year LRFS (80.9% vs. 67.3%, *P* < 0.001; Figure 1C) than patients with elevated ApoA-I level at diagnosis. In addition to ApoA-I level, sex, age, clinical stage, T stage, N stage, TC, and HDL-C were significantly associated with DSS, DMFS, and LRFS.

**Table 2:** Univariate analysis of prognostic factors for patients with nasopharyngeal carcinoma

Factor	5-year disease-specific survival (%)	5-year distant metastasis-free survival (%)	5-year locoregional recurrence-free survival (%)
Gender			
Male	76.8	78.9	76.0
Female	86.9	87.2	87.0
*P* value	**0.003**	**0.004**	**0.001**
Age (years)			
≤45	84.3	84.5	82.8
>45	74.6	77.5	74.7
*P* vallue	**<0.001**	**<0.001**	**<0.001**
Clinical stage			
I/II	94.8	94.4	92.6
III/IV	75.7	77.6	75.3
*P* value	**<0.001**	**<0.001**	**<0.001**
T status			
T1/T2	90.1	90.6	87.7
T3/T4	74.3	76.2	74.4
*P* value	**<0.001**	**<0.001**	**<0.001**
N status			
N0/N1	83.1	84.8	82.3
N2/N3	73.9	75.2	73.4
*P* value	**<0.001**	**<0.001**	**0.001**
VCA-IgA			
<1:80	86.3	85.5	87.8
≥1:80	78.7	80.8	78.2
*P* value	**0.024**	0.179	**0.011**
EA-IgA			
<1:10	83.2	80.1	82.9
≥1:10	77.9	80.4	77.6
*P* value	0.097	0.934	0.102
Triglyceride (mmol/L)			
<1.865	78.7	79.7	78.0
≥1.865	82.9	87.0	82.4
*P* value	0.201	0.130	0.285
Cholesterol (mmol/L)			
<5.045	78.1	78.5	76.4
≥5.045	80.9	83.7	81.4
*P* value	**0.043**	**0044**	**0.019**
HDL-C (mmol/L)			
<1.065	74.2	75.6	74.2
≥1.065	81.5	83.1	80.6
*P* value	**<0.001**	**0.005**	**0.003**
LDL-C (mmol/L)			
<5.715	79.3	80.8	78.7
≥5.715	91.7	92.3	84.6
*P* value	0.107	0.220	0.418
ApoA-I (g/L)			
≥1.025	81.3	83.4	80.9
<1.025	69.3	67.4	67.3
*P* value	**<0.001**	**<0.001**	**<0.001**
Apo-B (g/L)			
<1.880	79.3	88.9	78.8
≥1.880	88.9	80.9	77.8
*P* value	0.313	0.489	0.937
Treatment			
CRT	77.2	79.0	77.1
RT	84.6	85.4	82.8
*P* value	0.052	**0.023**	**0.031**

Abbreviations: VCA-IgA, immunoglobulin A/virus capsid antigen; EA-IgA, immunoglobulin A/early antigen; HDL-C, high-density lipoprotein cholesterol; LDL-C, low-density lipoprotein cholesterol; ApoA-I, apolipoprotein A-I; Apo-B, apolipoprotein B; CRT, chemoradiotherapy; RT, radiotherapy.

### Multivariate analysis of ApoA-I as an independent prognostic factor for DSS, DMFS, and LRFS

As shown in Table 3, age, sex, clinical stage, T stage, N stage, ApoA-I, TC, HDL-C and treatment were included in the multivariate analysis. Decreased serum ApoA-I level was a significantly independent predictor for the worse prognostic measures, including DSS [hazard ratio (HR) = 1.629, 95% confidence interval (CI) = 1.227–2.163; *P* = 0.001], DMFS (HR = 1.888, 95% CI = 1.370–2.603; *P* < 0.001), and LRFS (HR = 1.750, 95% CI = 1.278–2.396; *P* < 0.001). The advanced T and N stages were also independent indicators for inferior DSS, DMFS, and LRFS. In addition, younger age was an independent factor for superior DSS, DMFS, and LRFS.

**Table 3:** Multivariate Cox proportional hazards analysis prognostic factors in non-metastatic NPC patients

Variable	Death		Recurrence		Metastasis	
	HR (95% CI)	*P* value	HR (95% CI)	*P* value	HR (95% CI)	*P* value
Age, >45 vs. ≤45 years	1.939 (1.548–2.429)	**<0.001**	1.660 (1.295–2.128)	**< 0.001**	1.680 (1.300–2.171)	**<0.001**
Sex, male vs. female	1.269 (0.962–1.674)	0.0910	1.499 (1.085–2.072)	**0.014**	1.372 (0.987–1.909)	0.060
Clinical stage, IV/III vs. II/I	2.131 (1.207–3.759)	**0.009**	1.789 (0.979–3.268)	0.059	1.746 (0.884–3.447)	0.108
T stage, T4/T3 vs. T2/T1	2.041 (1.419–2.936)	**<0.001**	1.715 (1.154–2.549)	**0.008**	2.330 (1.507–3.603)	**<0.001**
N stage, N3/N2 vs. N1/N0	1.472 (1.164–1.862)	**0.001**	1.415 (1.084–1.848)	**0.011**	1.652 (1.263–2.163)	**<0.001**
ApoA-I (g/L), < 1.025 vs. ≥1.025	1.629 (1.227–2.163)	**0.001**	1.750 (1.278–2.396)	**<0.001**	1.888 (1.370–2.603)	**<0.001**
Cholesterol (mmol/L), <5.045 vs. ≥5.045	1.198 (0.960–1.495)	0.109	1.257 (0.979–1.613)	0.073	1.196 (0.925–1.545)	0.172
HDL-C (mmol/L), <1.065 vs. ≥1.065	0.899 (0.696–1.160)	0.411	0.984 (0.739–1.311)	0.914	1.010 (0.749–1.362)	0.947
Treatment, CRT vs. RT	1.421 (1.091–1.851)	0.009	1.241 (0.911–1.689)	0.171	1.337 (0.975–1.834)	0.071

Abbreviations: ApoA-I, apolipoprotein A-I; HDL-C, high-density lipoprotein cholesterol.

**Figure 2:**
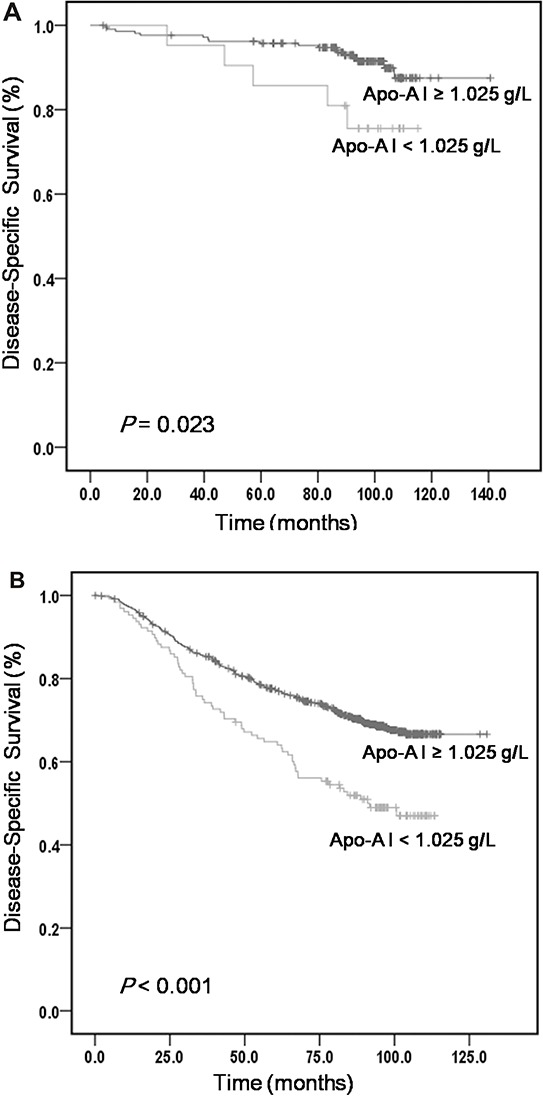
The five-year DSS analysis comparing serum levels of ApoA-I within different clinical stages **A.** DSS curves based on ApoA-I levels in stage I/II. **B.** DSS curves based on ApoA-I levels in stage III/IV.

We further classified patients within each stage level into two risk stratification groups based on ApoA-I level (Table 4). After stratification by clinical stages, ApoA-I remained a clinically and statistically significant predictor of prognosis (Figure 2).

**Table 4:** Five-year DSS analysis comparison of different levels of ApoA-I within each clinical stage

Clinical stage and ApoA-I level	No. of patients	5-year DSS (%)	*P* value
I/II			
ApoA-I ≥ 1.025 g/L	208	96.1	**0.023**
ApoA-I < 1.025 g/L	25	84.0	
III/IV			
ApoA-I ≥ 1.025 g/L	803	77.4	**<0.001**
ApoA-I < 1.025 g/L	160	67.0	

Abbreviations: DSS, disease-specific survival; ApoA-I, apolipoprotein A-I.

## DISCUSSION

This is believed to be the first large cohort study to evaluate the prognostic significance of serum biomakers of lipid metabolism including triglyceride, cholesterol, HDL-C, LDL-C, ApoA-I, and Apo-B in non-metastatic NPC patients. The results suggested that elevated serum ApoA-I was significantly associated with superior prognosis. Serum ApoA-I level segregated patients with NPC at each level of clinical stages into two significantly distinguished risk groups. Thus, the level of serum ApoA-I could complement clinical staging for risk definition and could be useful in selecting optimal treatment for patients with NPC at different stage.

ApoA-I, which belongs to the apolipoprotein A1/A4/E family, is the main polypeptide of human plasma HDL and plays a key role in cholesterol homeostasis [21]. Synthesized in the liver and small intestine, ApoA-I reverses transport of cholesterol from tissue to the liver for excretion, transfers fatty acids and ethanolamine back to cells for reuse, and acts as a cofactor for lecithin cholesterol acyltransferase to convert cholesterol to cholesterylester [22].

Accumulating studies have suggested a strong link between ApoA-I and different types of cancer. Similarly, our study showed that elevated ApoA-I level was significantly associated with better prognosis, independent of other variables in patients with non-metastatic NPC.

The biological mechanism underlying the relationship between ApoA-I level and cancer development remains to be clarified. NPC has long been associated with EBV [23]. A previous study has suggested that EBV-encoded LMP1 mediates interleukin-6 production in epithelial cells [24]. It is reported that interleukin-6 can stimulate hepatic production and secretion of secretory nonpancreatic phospholipase A2 (sPLA2), an acute-phase protein that is increased during infection and inflammation [25]. Overexpression of sPLA2 in transgenic mice has been reported to decrease the level of ApoA-I [26, 27]. Thus, the decrease in the ApoA-I level might be due to the subsequent stimulation of acute-phase proteins by EBV infection [19]. Moreover, the systemic inflammatory responseis suggested to promote tumor metastasis and progression. A recent study has revealed a potent immunomodulatory role for ApoA-I in the tumor microenvironment, altering tumor-associated macrophages from a pro-tumor to an anti-tumor phenotype [28]. Therefore, elevated levels of ApoA-I may indicate cancer-related inflammation and predict better prognosis. Additionally, ApoA-I has been identified as a prostacyclin (PGI2)-stabilizing factor and thus may have an anticlotting effect [28]. PGI2 is reported to act as a powerful anti-metastatic agent against melanoma cells, which may result from the inhibitory effect of PGI2 on platelet aggregation. In addition, inhibitors of PGI2 synthesis may increase metastasis [29]. This suggests that the anti-tumor effects of elevated ApoA-I levels might be mediated by stabilizing PGI2. Further investigation is required to provide a better understanding of these mechanisms.

Prognostic assessment is crucial for formation of appropriate treatment choices. Various prognostic factors for NPC have been identified and evaluated retrospectively. Most of these factors were identified by immunohistochemical staining of tumor tissue, such as survivin and livin [30]. However, few of these markers are currently used in routine clinical practice. Currently, the TNM staging system is the key prognostic determinant for patients with NPC. However, large variations in the clinical outcomes of patients with the same cancer stage have been reported [31]. In our study, elevated serum ApoA-I level was predictive of superior survival in patients with NPC independent of TNM stage. Therefore, serum ApoA-I could potentially allow clinicians to identify candidates for aggressive therapy and improve treatment outcomes. Patients classified with the same TNM stage might be stratified into different disease recurrence-risk groups based on serum ApoA-I level, and thus treated with systemic approaches of different intensities. For example, in patients with early-stage disease (stages I/II) and low ApoA-I levels, CRT might be more beneficial than RT alone. However, for patients with advanced-stage disease (stages III/IV) and low ApoA-I levels, the current chemotherapy regimen might be insufficient, and a more intensive chemotherapy regimen or targeted therapy may be needed.

Our study had two main strengths: (i) all patients were newly diagnosed, which ruled out any impact on patient outcome of any possible disproportionate pretreatment; and (ii) relatively long follow-up period. However, our study also had two main limitations: (i) because of its retrospective nature, the selection bias could not be completely eliminated; and (ii) this was a retrospective study restricted to one local hospital. Therefore, a prospective study with a large number of cases is needed to confirm a correlation between ApoA-I and NPC prognosis.

In conclusion, this study suggested that the serum level of ApoA-I at diagnosis is a prognostic indicator of clinical outcome in non-metastatic NPC and may complement clinical staging for risk definition. Future prospective clinical studies are required to confirm our findings.

## MATERIALS AND METHODS

### Patients and data collection

We retrospectively reviewed the medical records of 1196 patients with newly diagnosed non-metastatic NPC between January 2004 and March 2007 at the Sun Yat-sen University Cancer Center, China. The criteria for case inclusion were as follows: (i) pathologically diagnosed non-metastatic NPC; (ii) Karnofsky Performance Status score ≥70; (iii) no previous treatment; and (iv) adequate clinical information and follow-up data. Patients were excluded if: (i) they were taking hormone replacement therapy or any drugs known to affect lipid metabolism; or (2) if they had previous malignancy or a second primary tumor. Each patient underwent a pre-therapeutic testing, including physical and neurological examinations, hematological and biochemistry profiles, endoscopic examination of the nasopharynx, computed tomography or magnetic resonance imaging of the head and neck, chest radiography, abdominal ultrasonography, emission computed tomography, or positron emission tomography. Clinical staging was reclassified according to the criteria of the 2002 Union International Control Cancer staging system. All pathological specimens were reviewed and reclassified by central review according to the World Health Organization criteria for pathologic diagnosis. This study was approved by the Institutional Review Board of Sun Yat-sen University Cancer Center. Informed consent for the collection of medical information was provided at the first visit of all patients.

The clinical data were collected at diagnosis, including patient demographics, Karnofsky Performance Status, the serum level of lipids and lipoproteins, and the serum antibody titers of Epstein-Barr virus (EBV) immunoglobulin A/virus capsid antigen (VCA/IgA) andimmunoglobulin A/early antigen (EA/IgA). Serum levels of triglyceride, cholesterol, HDL-C, low-density lipoprotein cholesterol (LDL-C), ApoA-I, and apolipoprotein B (Apo-B) were examined in early morning samples obtained before therapy and immediately measured using a Hitachi 7600–020 automatic biochemical analyzer (Hitachi High-Technologies, Tokyo, Japan).

### Treatment and follow-up

Patients were treated with different regimens based on their clinical characteristics in line with the clinical practice guidelines for NPC in the Sun Yat-sen University Cancer Center and National Comprehensive Cancer Network guidelines. According to the clinical practice guidelines during the study period, RT alone was used for patients in stages I–IIA, radiation with concurrent platinum-based chemotherapy was used for those in stage IIB, and concurrent CRT, with or without neoadjuvant or adjuvant chemotherapy, was used for those in stages III–IV. For RT, megavoltage photons (6 MV) were used to treat the primary tumor and neck lymph nodes. RT was given five times a week at 2 Gy/day. The median radiation dose was 70 Gy (range, 60–80 Gy) to the nasopharyngeal region and 60 Gy (range, 40–72 Gy) to the initially involved cervical node. In 41 patients with stage I disease, 38 (92.7%) were treated with radiotherapy alone, 3 (7.3%) were treated with cisplatin and 5-FU based neoadjuvant (2 patients) and concurrent chemotherapy (1 patient). In193 patients with stage II disease, 130 (67.4%) were treated with radiotherapy alone and 63 (32.6%) were treated with cisplatin and/or 5-FU based neoadjuvant (13 patients) and/or concurrent chemotherapy (50 patients). In 608 patients with stage III disease, 136 (22.4%) were treated with radiotherapy alone and 472 (77.6%) were treated with cisplatin and/or 5-FU based neoadjuvant (261 patients) and/or concurrent chemotherapy (291 patients). In 354 patients with stage IV disease, 30 (8.5%) were treated with radiotherapy alone, 324 (91.5%) were treated with cisplatin and/or 5-FU based neoadjuvant (206 patients) and/or concurrent chemotherapy (200 patients). Neoadjuvant chemotherapy consisted of cisplatin with 5-fluorouracil every 3 weeks for two or three cycles. Concurrent chemotherapy that consisted of cisplatin was administered on days 1, 22, and 43 of RT.

After treatment was completed, patients were followed up every 3 months in the first 3 years and every 6 months thereafter. The last follow-up date was December 31, 2014 for all the available patients.

### Statistical analysis

Statistical analysis was performed using SPSS version 18.0 (SPSS Inc., Chicago, IL, USA). The major endpoints used for assessment in the current analysis were DSS, DMFS, and LRFS. We calculated DSS from diagnosis to the date of death from disease-related causes or the date of the last follow-up visit; DMFS to the first distant relapse or the date of the last follow-up visit; and LRFS to the first locoregional relapse or the date of the last follow-up visit.

Receiver operating characteristic (ROC) curves were used to select the most appropriate cutoff points of serum lipids and lipoproteins to stratify patients at a high risk of malignancy-related death. The score closest to the point with both maximum sensitivity and specificity was selected as the cutoff value. The χ^2^ test was used to compare baseline clinical characteristics between different serum levels of ApoA-I. The DSS, DMFS, and LRFS rates were calculated by the Kaplan-Meier method, and survival was compared using the log-rank test. Significant variables in the univariate analysis were considered as variables for the multivariate analysis of survival. The later was performed by the Cox proportional hazards model. *P* < 0.05 was considered statistically significant. All *P* values corresponded to two-sided significance tests.

## SUPPLEMENTARY FIGURE


