# Supplementary material for: Serum apolipoprotein A-I is a novel prognostic indicator for non-metastatic nasopharyngeal carcinoma
Source: Oncotarget. 2015 Oct 19;6(41):44037–48. doi: 10.18632/oncotarget.5823 (PMC4791285; doi:10.18632/oncotarget.5823)
Supplement: Supplementary file 1 [file oncotarget-06-44037-s001.pdf]

SUPPLEMENTARY FIGURE

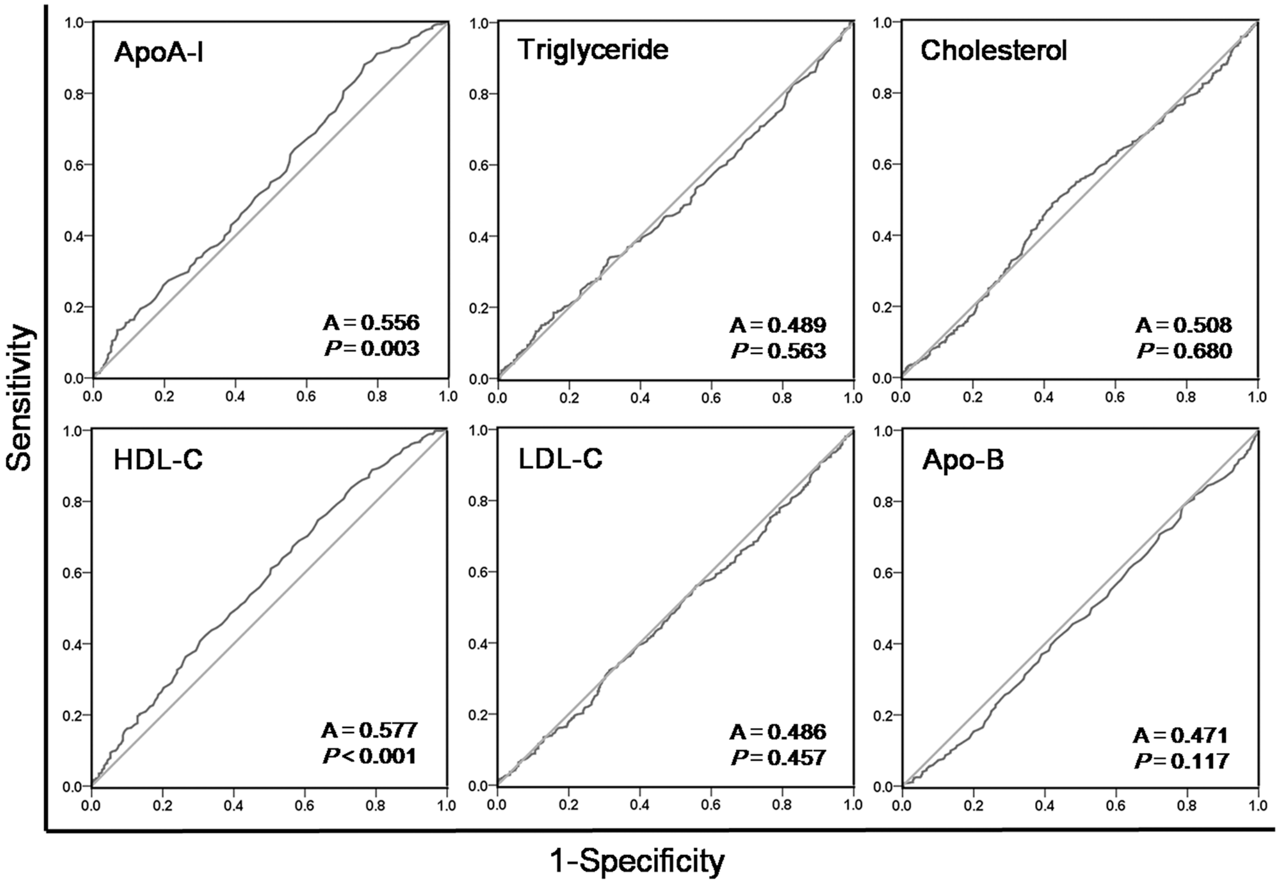

Supplementary Figure S1: **Receiver operating characteristic (ROC) curves of baseline serum levels of lipid and lipoprotein.**
